# Egyptian Novel Goose Parvovirus in Immune Organs of Naturally Infected Ducks: Next-Generation Sequencing, Immunohistochemical Signals, and Comparative Analysis of Pathological Changes Using Multiple Correspondence and Hierarchical Clustering Approach

**DOI:** 10.3390/v17010096

**Published:** 2025-01-13

**Authors:** Mohamed A. Lebdah, Amal A. M. Eid, Reham M. ElBakrey, Abd Elgalil. El-Gohary, Mohamed G. Seadawy, Mohamed R. Mousa, Hagar F. Gouda, Nehal I. A. Goda, Mostafa F. El-Hosseny, Ahmed S. El-tahlawy, Rokayya Sami, Rasha A. Al-Eisa, Sarah S. Helal

**Affiliations:** 1 Department of Avian and Rabbit Medicine, Faculty of Veterinary Medicine, Zagazig University, Zagazig 44511, Egypt; lebdahmohamed@gmail.com (M.A.L.); rehamemara3@gmail.com (R.M.E.); 2 Department of Poultry and Rabbit Diseases, Faculty of Veterinary Medicine, Kafrelsheikh University, Kafrelsheikh 33511, Egypt; abdelgalilelgohary@gmail.com; 3 Biodefense Center for Infectious and Emerging Diseases, Ministry of Defense, Cairo 11775, Egypt; biologist202054@yahoo.com (M.G.S.); mfm.memo@gmail.com (M.F.E.-H.); 4 Faculty of Biotechnology, October University for Modern Sciences & Arts (MSA University), 6th of October City 12451, Egypt; 5 Department of Pathology, Faculty of Veterinary Medicine, Cairo University, Giza 12211, Egypt; mohamed.refat@cu.edu.eg; 6 Department of Animal Wealth Development (Biostatistics Subdivision), Faculty of Veterinary Medicine, Zagazig University, Zagazig 44511, Egypt; hagarfathy@zu.edu.eg; 7 Department of Histology and Cytology, Faculty of Veterinary Medicine, Zagazig University, Zagazig 44511, Egypt; nihalgoda91@gmail.com; 8 Food Hygiene, Safety, and Technology Department, Faculty of Veterinary Medicine, Zagazig University, Zagazig 44511, Egypt; aseltahlawy@vet.zu.edu.eg; 9 Department of Food Science and Nutrition, College of Sciences, Taif University, P.O. Box 11099, Taif 21944, Saudi Arabia; rokayya.d@tu.edu.sa; 10 Department of Biology, College of Sciences, Taif University, P.O. Box 11099, Taif 21944, Saudi Arabia; r.hasan@tu.edu.sa

**Keywords:** novel goose parvovirus, immunohistochemistry, multiple correspondence, hierarchical clustering, next-generation sequencing, Egypt, immune system organs, ducks

## Abstract

The present study aims to better understand the nature of currently circulating GPV strains and their pathological impact on the immune system during natural outbreaks among different duck breeds in Egypt. For this purpose, 99 ducks (25 flocks) of different breeds, aged 14–75 days, were clinically examined, and 75 tissue pools from the thymus, bursa of Fabricius, and spleen were submitted for virus detection and identification. Clinical and postmortem findings were suggestive of GPV infection. Concerning the immune system organs, atrophy in the thymus (60.6%), bursa (45.5%), and spleen (38.3%) was the most common gross lesion. Microscopically, the pathological impact of the virus was exhibited by a necrotic thymic cortex with Hassall’s corpuscle disintegration, the disappearance of normal bursal histological morphology accompanied by atrophied follicles and lymphocytic depletion, and apoptosis of B-lymphocytes in lymphoid follicles of the spleen. Furthermore, immunohistochemical examination revealed positive signals of the parvovirus detected in thymic lymphocytes in the cortex, bursa-dependent lymphoid follicle of the medulla, and diffuse positive expression of viral antigens in the spleen. GPV was detected in ducks using polymerase chain reaction, with the highest percentage of positive detection in the bursa of Fabricius (76%). Next-generation sequencing and phylogenetic analysis revealed that the detected virus was a variant of GPV, globally named novel GPV (NGPV), and closely related to Chinese NGPV isolates. To our knowledge, the current study is pioneering to address the immunopathological impact of NGPV among naturally infected ducks confirmed with full genome sequencing and immunohistochemical identification worldwide.

## 1. Introduction

Parvovirus infection has frequently been associated with gastrointestinal disease in a wide range of hosts and has been described in different species of birds, including waterfowls [1,2]. Diseases caused by waterfowl parvoviruses (WFPVs) cause serious economic losses in regions with intensive goose and duck production [3,4,5].

Waterfowl parvovirus infection was first detected in China and described by Fang in 1956 [6], primarily known and investigated by Derzsy and his team in Hungary in 1967. It was named after him, and described in goslings as a highly fatal disease characterized by anorexia, prostration, and death within 2 to 5 days [7]. This disease is caused by goose parvovirus (GPV) [8].

During the late 1980s, a new disease called Muscovy 3-week disease, caused by a related Muscovy duck parvovirus (MDPV), appeared in Muscovy ducks (*Cairina moschata*) worldwide. This disease is characterized by mortality of between 10 and 80%, neurological, locomotor, and enteric signs, and surviving ducks permanently suffer from abnormal feather development and growth retardation [9].

Generally, Pekin (*Anas platyrhynchos*) and Mule (a sterile hybrid cross between the Muscovy and Pekin breeds) ducks are resistant to classical WFPV infections. A new disease known as short beak and dwarfism syndrome (SBDS) has been reported in Mule and Pekin ducks in several countries, including France, Poland, and China. SBDS is characterized by strong growth retardation, beak atrophy, and tongue protrusion [10,11,12]. Based on virus isolation and molecular identification, a variant strain of GPV named novel goose parvovirus (NGPV) was identified as the causative agent of SBDS in ducklings, and it has been circulating since 2015 in China [13].

WFPVs’ genome is represented by approximately 5100 nucleotides of long linear single-stranded DNA, with an icosahedral symmetry and a diameter of 20–22 nm. Two main open reading frames (*ORFs*) are identified. The 5′ *ORF* (*ORF1*) encodes nonstructural proteins (*NS*), which are involved in viral replication, transcription, and assembly. The 3′ *ORF* (*ORF2*) encodes capsid viral proteins (Caps), including the structural proteins *VP1*, *VP2*, and *VP3*, which play important roles in tissue tropism, host range, and pathogenicity. WFPVs can be antigenically categorized into GPV-related or MDPV-related groups based on their nucleotide sequences [14,15]. NGPVs isolated from SBDS cases belong to either the West European subgroup or the Chinese subgroup of the GPV-related group, as they share a strong antigenic relationship with classical GPV strains. Furthermore, genome comparison showed that the sequence similarity between NGPVs and GPVs (90% to 98%) was greater than that between NGPVs and MDPVs (78.6% to 85.0%). Therefore, it can be regarded as a variant GPV [13,16,17,18].

Capsid proteins play an important role in virus host range, tropism, pathogenicity, and protective immune response, as they contain viral antigenic sites. These three capsid proteins, *VP1*, *VP2*, and *VP3,* constitute the icosahedral capsid in a ratio of approximately 1:1:8, respectively [19,20]. Consequently, they are utilized for discriminatory diagnostic tools, epidemiological monitoring of WFPVs, and the selection and evaluation of protective vaccines [21,22,23,24].

Owing to the difficulties facing WFPV isolation, conventional PCR technology has been used as the main screening and diagnostic tool not only for the detection of WFPVs but also for the differentiation between MDPVs and GPVs [25,26]. Recently, TaqMan-based real-time PCR has gained wide acceptance due to its high specificity and sensitivity [27,28]. As a result of high antigenic homology, especially between classical GPV and NGPVs, sequence-independent techniques such as next-generation sequencing (NGS) have allowed the confirmatory identification and differentiation between NGPVs and GPVs [1,29]. Immunohistochemistry (IHC) has been used to detect parvovirus antigens in various organs [30,31].

Since 2019, SBDS outbreaks have been frequently recorded in different localities in Egypt among commercial Pekin and Mule flocks, leading to drastic economic losses for farm owners. The causative agent was isolated on primary duck embryo liver (DEL) cell culture, detected using conventional PCR, and identified by partial genome sequencing and phylogenic analysis of the *VP1* gene as a variant GPV (NGPV) that clustered with the Chinese NGPVs in the same group [32].

Most studies have investigated the negative impact of classical and variant GPVs on the digestive system and accessory organs [31,33], including our own original research [34], with less focus on the immune system [30,35]. In the present study, this relatively rare information motivated us to investigate GPV pathogenesis among immune system organs in naturally occurring outbreaks in Egypt and the full genome sequencing of three Egyptian isolates using next-generation sequencing.

## 2. Materials and Methods

### 2.1. Ethics Considerations

Animal handling and sample collection were reviewed and approved by the ZU-IACUC committee at Zagazig University in Egypt (approval number: ZUIACUC/2/F/176/2022; approval date: 28 September 2022).

### 2.2. Clinical, Postmortem Examination and Sample Collection

A total of 99 diseased ducks, representing 25 flocks from different breeds (15 Pekin flocks, 7 Muscovy flocks, 2 Native flocks, and 1 Mule flock), ranging in age from 14 to 75 days, were collected from different localities in Sharkia Province, Egypt. All ducks hatched from vaccinated breeders, except for the Native flocks. The descriptive data of clinically examined duck flocks is shown in (Supplementary Table S1). The study design, field assessment of the current state of parvovirus infections in ducks, and sample collection were carried out during a three-year period (2021–2023). The collected ducks, suspected of having a parvovirus infection, were subjected to clinical and postmortem examinations.

Regarding collecting samples for virus detection, 75 tissue pools from the immune system organs of those 25 diseased duck flocks (3 tissue pools per each flock from thymus, bursa of Fabricius, and spleen, and each pool collected from 2 to 3 ducks per flock) were harvested separately per organ (25 thymus tissue pools, 25 bursa of Fabricius tissue pools, and 25 spleen tissue pools) and stored at approximately 4 °C in leak-proof containers with ice and preserved at −80 °C for molecular detection.

Representative samples from each flock were prepared as 10% tissue suspension, inoculated into embryonated chicken eggs (ECE) via the allantoic cavity route, and candled daily for seven days [33]. The allantoic fluid was then harvested and tested using the rapid hemagglutination test. So, the absence of any mixed infection with hemagglutinating (HA) agents was confirmed.

### 2.3. Histopathological and Immunohistochemical (IHC) Examination

Tissue specimens from the thymus, spleen, and bursa of Fabricius were collected from all examined birds, preserved in neutral buffered formalin 10%, and routinely processed, sectioned, and stained with hematoxylin and eosin (H&E) [36]. Tissue sections were examined using a Leica DM4 B light microscope (Leica, Germany) and captured using a Leica DMC 4500 digital camera (Leica, Wetzlar, Germany) linked to LAS-X software (Leica, Wetzlar, Germany, https://www.leica-microsystems.com/products/microscope-software/p/leica-las-x-ls/, accessed on 9 September 2024).

For hyperimmune serum production, four New Zealand rabbits were vaccinated with DEPARMUNE^®^ inactivated oil emulsion vaccine (Ceva-Phylaxia, Budapest, Hungary, Batch No: 001KG1D) against waterfowl parvovirus via a series of injections (0.5 mL) following the schedule described by Samiullah et al. (2006) [37]. Blood samples were collected and centrifuged to separate serum. IgGs were precipitated using ammonium sulfate [38]. Purified IgG was used as the primary antibody at a dilution of 1:500 in phosphate-buffered saline (PBS) [38].

IHC was performed as described by Mesalam et al. (2021) [39]. The retrieval of heat-induced antigen was conducted in a microwave oven for 15 min using Tris-EDTA buffer (10 mM Tris-base, 1 mM EDTA solution, 0.05% Tween 20, pH 9). Tissue slides were washed with phosphate-buffered saline (PBS). The endogenous peroxidase blocking step was performed by adding three drops of 3% H_2_O_2_ (Bio-SB, Goleta, CA, USA) to tissue sections and incubating for 10 min. Tissue slides were incubated with the primary antibodies (rabbit anti-waterfowl parvovirus, 1:500 in PBS) for 2 h in a humidity chamber, followed by washing with PBS and incubation with goat anti-rabbit HRP-labelled secondary antibodies (Abcam, Cambridge, UK) for 2 h at room temperature. Finally, a DAB Substrate Kit (Abcam, Cambridge, UK) was used for substrate detection. As a negative control, the primary antibody was replaced by negative serum on parvovirus-infected immune tissue specimens. Additionally, normal non-infected duck immune tissues were incubated with the primary anti-waterfowl parvovirus antibodies [40].

### 2.4. DNA Extraction, Conventional and TaqMan Real-Time PCR Amplification

DNA was extracted from the bursa of Fabricius, thymus, and spleen tissue pools using the GeneJET Genomic DNA Purification Kit (Thermo Fisher Scientific™, Baltics UAB, Lithuania, Catalogue No: K0721) according to the manufacturer’s instructions. DNA was quantified using a NanoDrop spectrophotometer (Thermo Fisher Scientific, Waltham, MA, USA).

Oligonucleotide primers (TIB MOLBIOL, Berlin, Germany) used for conventional PCR and real-time PCR are shown in (Table 1).

Using conventional PCR assay, 27 tissue pools from Muscovy and Native flocks were identified by using the DreamTaq PCR Master Mix (2×) kit in accordance with the manufacturer’s recommendations (Thermo Fisher Scientific™, Baltics UAB, Lithuania, Catalogue No: K1071) and performed twice for each sample. A total of 50 μL of each PCR mix was optimized as follows: 25 µL of 2× DreamTaq Green PCR Master Mix, 1 µL forward primer (10 μmol/L each), 1 µL reverse primer (10 μmol/L each), 5 µL DNA template, and 18 µL nuclease-free water. The thermal cycling conditions were optimized as follows: one cycle of 95 °C 1 min (initial denaturation), followed by 40 cycles of 95 °C for 30 s, 60 °C for 30 s, 72 °C for 1 min, and a final extension step at 72 °C for 10 min. The amplification products were subjected to 1% agarose gel electrophoresis using a ready-to-use GeneRuler 1 kb DNA Ladder (Thermo Fisher Scientific™, Baltics UAB, Lithuania, Catalogue No: SM0313).

Using the real-time PCR technique, 48 tissue pools from Pekin and Mule flocks were detected. A total of 25 μL of PCR mix per sample was done by adding 12.5 μL of Brilliant Multiplex QPCR Master Mix (Agilent technologies, Santa Clara, CA, USA, Catalog no: 600553), 0.5 μL of each primer (10 μmol/L each), 1 μL of probe (10 μmol/L), 3 μL of DNA template, and nuclease-free water in an amount to adjust the total reaction volume to 25 μL. The thermal profile was set as: 1 cycle at 95 °C for 10 min, followed by 40 cycles at 95 °C for 15 s, 58 °C for 1 min, and 72 °C for 30 s using the AriaMx Real-time PCR System (Agilent Technologies, Santa Clara, CA, USA). Positive real-time PCR samples were resubmitted to discriminative conventional PCR to detect any mixed infection with MDPV.

### 2.5. Multiple Correspondence Analysis (MCA) and Hierarchical Clustering Analysis (HCA)

Multiple correspondence analysis (MCA) was performed to summarize and visualize the data and assess the associations between the presence of various gross lesions and microscopic features in different breeds and ages of the examined ducks. Hierarchical clustering analysis (HCA) was used in combination with MCA to profile and group the ducks or variables with similar characteristics. MCA and HCA provided convenient and easy-to-interpret analytical tools for assessing categorical data relationships [41] and were performed using the “factoMineR” package R-version 3.4.3 (R Development Core Team, Vienna, Austria).

### 2.6. Next-Generation Sequencing (NGS)

Library preparation was done as follows: viral DNA fragmentation into smaller pieces with adapter ligation to its end. These adapters contained sequences that were recognized by the Illumina sequencing platform and allowed for amplification and sequencing of the DNA fragments using the Nextera XT DNA Library Preparation Kit (Illumina, San Diego, CA, USA, Catalogue No: FC-131-1024). The prepared DNA library was loaded onto the Illumina MiSeq sequencer (Illumina, San Diego, CA, USA, Serial no: SY-410-1003). The sequencing process involved clustering of the DNA fragments on a flow cell, followed by the cyclic addition of fluorescently labeled nucleotides, which were incorporated into the growing DNA strands. The fluorescent signals generated during this process were detected using the sequencer and used to determine the sequence of each DNA fragment.

Raw sequencing data were processed and analyzed using the bacterial and viral bioinformatics resource center (BV-BRC) (bv-brc.org) and molecular evolutionary genetics analysis version.11 (MEGA.11) software and the data were submitted to the National Center for Biotechnology Information (NCBI) (https://www.ncbi.nlm.nih.gov/) and accessed on 9 September 2024. The phylogenetic tree was designated based on BLAST analysis of the three whole genome-sequenced isolates in the current study using the NCBI database. Up to 30 NGPV complete genome sequences, which were much closer to NGPV isolates in the present study, were selected to construct the phylogenetic tree using MEGA.11 software. The NCBI reference GPV virulent B strain (Accession no: NC_001701) was blasted with the three identified NGPV strains and inserted into the whole genome, capsid protein (*VP*), and nonstructural (*NS*) phylogenetic trees to determine the nucleotide diversity among them.

*VP* sequences of two European strains, the French Hoekstra vaccine strain (accession number: AY496907 for *VP1*/*VP2* and AY496894 for *VP3*), and the Hungarian LB vaccine strain (accession number: AY496900 for *VP1*/*VP2*, and AY496887 for *VP3*) [21] were blasted with the strains in the current study to determine the *VP* nucleotide homology. The viral genome was annotated and interpreted using the Vgas viral genome annotation system (http://guolab.whu.edu.cn/vgas, accessed on 9 September 2024) [42]. All procedures for molecular identification were performed according to the biosafety measures in the Biodefense Center for Infectious and Emerging Diseases, Ministry of Defense, Egypt.

## 3. Results

### 3.1. Clinical and Postmortem Examination

**Figure 1:**
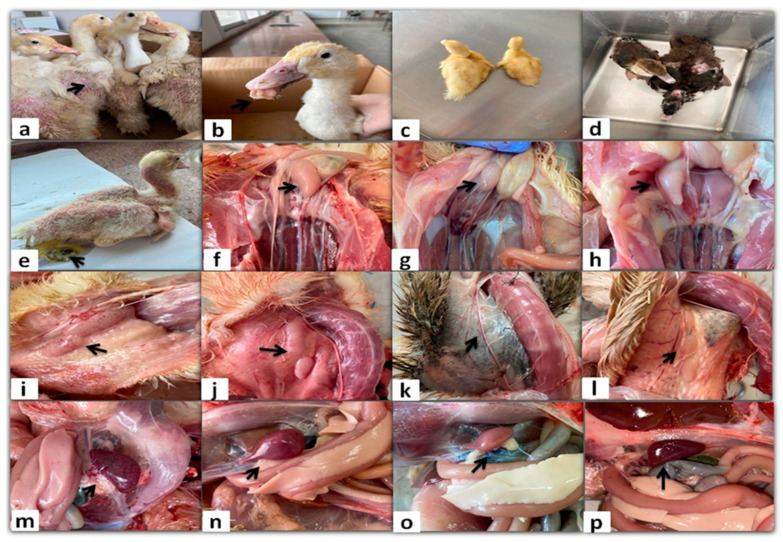
Clinical signs (**a**–**e**) and gross lesions (**f**–**p**) of naturally NGPV-infected flocks. Abnormal feathering and skin redness in the adult Pekin ducks (**a**). Short beak with protruding tongue in the Pekin duck (**b**). The growth retarded Pekin duckling compared to the normal one in the same flock (**c**). The growth retarded Muscovy duck compared to the normal one in the same flock (**d**). Whitish diarrhea, general weakness, and abnormal feathering in back and neck regions in the Muscovy duckling (**e**). Bursal enlargement (**f**) and bursal atrophy (**g**) compared to the normal one in the same Pekin flock (**h**). Thymus enlargement with hemorrhage (**i**) and Thymus congestion (**j**) in both adult Muscovy and Pekin ducks, respectively. Thymus atrophy (**k**) in the Muscovy duckling compared to the normal one in the same flock (**l**). Spleen mottling (**m**) and enlargement (**n**) in adult Muscovy and Pekin ducks, respectively. A pale atrophied spleen in the young Pekin duck (**o**) compared to the normal spleen in the same flock (**p**).

The morbidity rate ranged from 1.25 to 33% and reached up to 50% in two Pekin flocks, while the mortality rate did not exceed 2% and was recorded only in six flocks (three Pekin, two Muscovy, and one Mule). The examined ducks exhibited typical clinical signs, including growth retardation (*n* = 99/99; 100%), whitish diarrhea (*n* = 97/99; 98%), abnormal feathering and skin redness (*n* = 56/99; 56.6%), general weakness (*n* = 42/99; 42.4%), short beaks with protruding tongues (*n* = 52/99; 52.5%), or with non-protruding tongues (23/99; 23.2%) (Figure 1). The gross lesions of infected ducks in the current study included variable lesions in the organs of the immune system. Atrophy of the thymus, bursa of Fabricius, and spleen was the most prominent lesion, with a percentage of 60.6, 45.5, and 38.4, respectively (*n* = 60/99; 45/99; 38/99). In contrast, enlarged thymus with or without hemorrhage (*n* = 11/99; 11.1%), enlarged bursa (*n* = 13/99; 13.1%), and mottled or congested spleen (*n* = 17/99; 17.2%) were less common (Figure 1).

### 3.2. Histopathological and Immunohistochemical Findings

**Figure 2:**
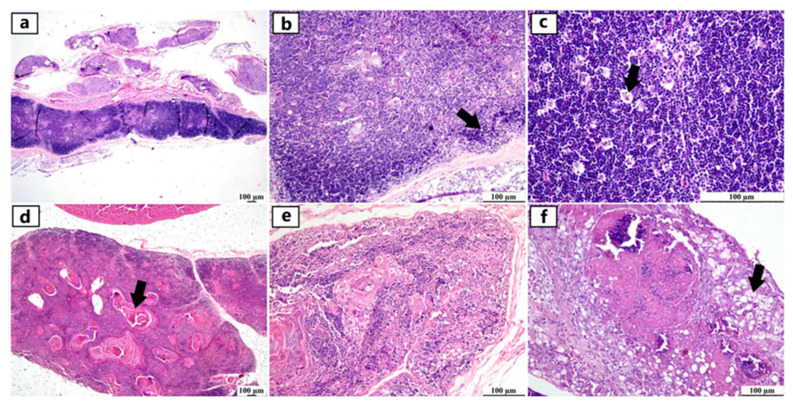
Photomicrographs of the thymus from different infected duck flocks (H&E). Young Pekin duck showed atrophy of the thymus gland (**a**). Young Pekin duck showed an atrophied thymus with a prominent decrease in cortical width, giving the appearance of increased medullary size (arrow) (**b**). Young Pekin duck showed lymphoid depletion in the thymic cortex, as demonstrated by remaining lymphocytes with pyknotic nuclei (necrotic/apoptotic cells) (arrow) (**c**). Adult Pekin duck showed accumulation of an abundant eosinophilic necrotic material in the thymic medulla (arrow) (**d**). Adult Pekin duck showed severe thymic atrophy illustrated by the presence of few lymphoid cells within this remnant of the thymus; thymic (Hassall’s) corpuscles are prominent in the medulla (**e**). Adult Pekin duck showed extensive damage and necrosis of the thymic gland with the total absence of demarcation between cortex and medulla in the thymic lobules, accumulation of necrotic debris, and vacuolation of epithelial cells (arrow) (**f**).

Examination of thymus sections revealed various histopathological alterations among several breeds of different ages. Thymic atrophy was commonly detected in several sections and characterized by a marked decrease in cortical width, resulting in an increased medullary size. Diffuse apoptosis of lymphocytes was demonstrated by the presence of scattered clear areas containing small dark nuclei, which is associated with hydropic degeneration and vacuolation of thymic epithelial cells. Thymic Hassall’s corpuscles appeared prominent in the medulla of a severely atrophied thymus, accompanied by an increased accumulation of necrotic granulocytic leukocytes. The absence of demarcation between the cortices and medullae was also observed (Figure 2).

**Figure 3:**
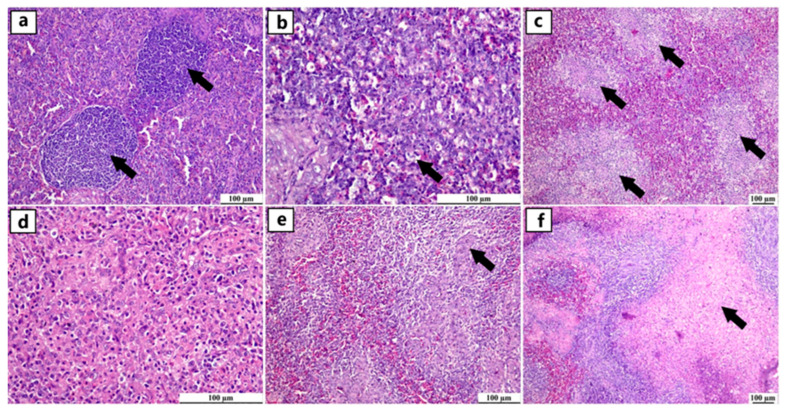
Photomicrographs of spleen from different infected duck flocks (H&E). Young Muscovy ducks with reactive lymphoid follicles (germinal centers), consisting of a nodular collection of large lymphoblast-like cells enclosed by a thin connective tissue capsule (arrows) (**a**). Young Pekin ducks with lymphocytic depletion illustrated by granular debris within the vacuolar spaces of apoptotic cells (arrow) (**b**). Young Pekin ducks showed severe loss of lymphocytes in white pulps and were replaced by fibrinous deposits (arrows) (**c**). Adult Pekin duck tissue sections at higher magnification displayed the paucity of lymphocytes in the white pulp (**d**). Young Muscovy ducks with obvious hyperplasia of ellipsoidal reticular cells around penicilliform capillaries (arrow) (**e**). Young Pekin ducks with infarcted areas separated by hemorrhagic margins (arrow) (**f**).

Microscopic examination of the spleen revealed a reactive spleen characterized by numerous reactive lymphoid follicles (germinal centers), which were composed of a nodular collection of large lymphoblast-like cells enclosed by a thin connective tissue capsule. Apoptosis of B-lymphocytes was detected in these follicles, as illustrated by the presence of granular materials within vacuolar spaces and the rapid proliferation of lymphocytes. Additionally, the grossly enlarged spleen manifested microscopically as hyperplasia of ellipsoidal reticular cells around the penicilliform capillaries. A sporadic case showed large infarcted zones separated from the surrounding splenic parenchyma with hemorrhagic margins (Figure 3).

**Figure 4:**
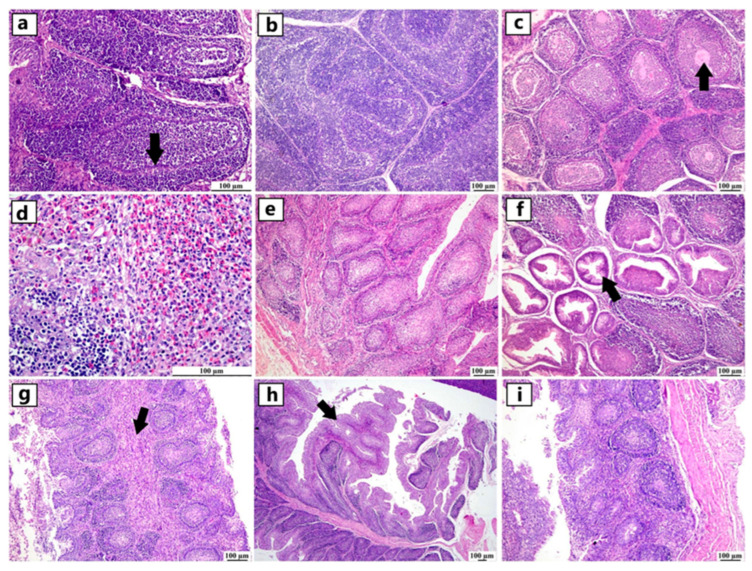
Photomicrographs of bursa of Fabricius from different infected duck flocks (H&E). Young Pekin ducks showed depletion of lymphoid follicles with prominent epithelial cells located between the cortex and medulla (arrow) (**a**). Adult Muscovy ducks showed reactive lymphoid follicles with increasable depleted lymphocytes, demonstrated by vacuolar spaces (**b**). Young Pekin ducks showed severely damaged follicles with cystic spaces in the medullary area containing proteinaceous exudates (arrow) (**c**). Adult Muscovy duck tissue sections at higher magnification showed an expansion of the interfollicular connective tissue with many heterophils and macrophages (**d**). Adult Pekin ducks with marked loss of lymphocytes in lymphoid follicles (**e**). Adult Pekin ducks showed collapsed plica, and mucosal epithelial cells started to fold into the damaged follicles; note that the epithelium cells appeared as ductal structures due to the sectioning plane (arrow) (**f**). Young Pekin ducks showed marked atrophied follicles and expansion of the interfollicular spaces with abundant fibroplasia and inflammatory cell aggregates (arrow) (**g**). Young Muscovy ducks with an extensive folding of plica epithelium (arrow) (**h**). Young Pekin ducks showed atrophied bursa with a marked reduction of bursa folds and a decreased number of lymphoid follicles (**i**).

Examination of the bursa of Fabricius showed depletion of lymphoid follicles associated with atrophied follicles and increased plica folds in severely affected flocks. The interfollicular connective tissue was markedly expanded, with edema, heterophils, and macrophage infiltration. Damaged follicles displayed cystic spaces surrounding the proteinaceous fluid. Due to severe lymphoid depletion, the zone of epithelial cells between the cortex and medulla appeared very prominent (Figure 4).

**Figure 5:**
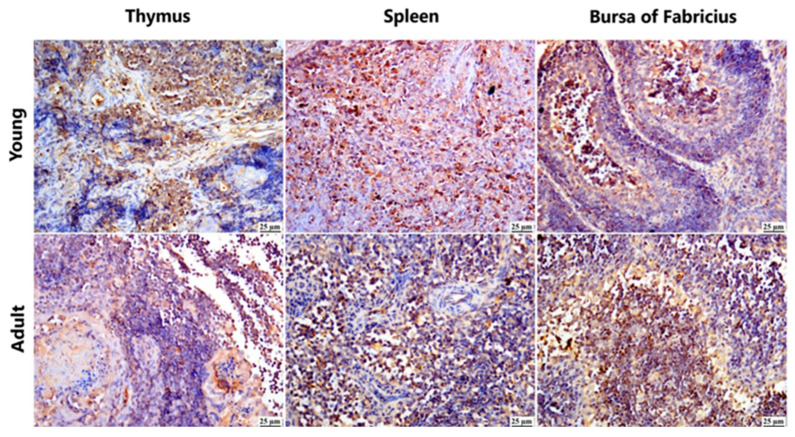
Photomicrograph demonstration of NGPV antigen in different organs of the immune system of various infected flocks (IHC). Positive expression was detected in the thymic lymphocytes in the cortex and, to a lesser extent, in the medulla. The spleen showed diffuse positive expression of viral antigen in the bursa-dependent lymphoid follicle and peri-ellipsoidal lymphoid sheath (PELS) of young and adult infected duck flocks. The bursal lymphoid follicles exhibited a positive reaction, demonstrating viral antigens mainly in the medullary lymphocytes.

Positive expression of viral antigens was detected in different organs of the immune system of several infected breeds at different ages, which suggests replication of the virus in lymphoid tissue with subsequent immune suppression (Figure 5).

**Figure 6:**
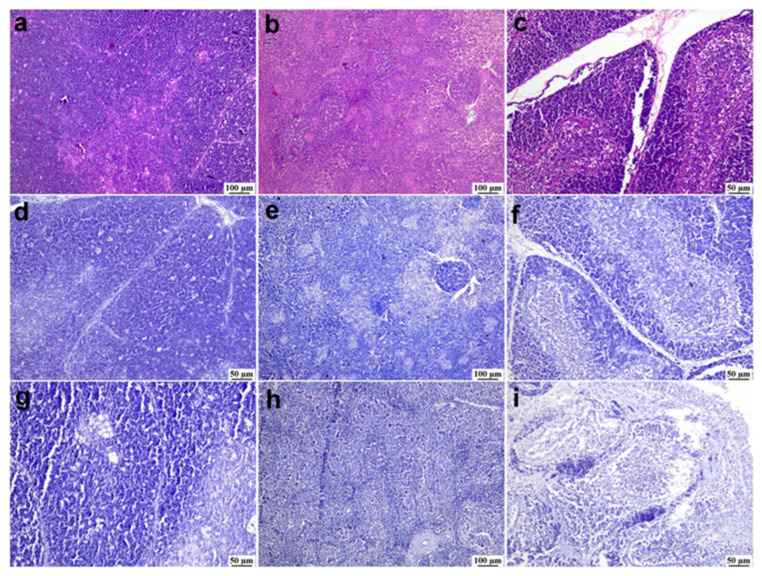
Photomicrographs of bursa, thymus, and spleen of non-infected ducks. (**a**–**c**) Normal histological structure of the thymus, spleen, and bursa of Fabricius from flocks free from NGPV infection, respectively (H&E). (**d**–**f**) Negative expression of the viral antigen in the thymus, spleen, and bursa of Fabricius of the non-infected flocks, respectively (IHC). (**g**–**i**) Negative expression of viral antigen in the thymus, spleen, and bursa of Fabricius of the infected flocks after the deletion of the primary antibody incubation step, respectively (IHC).

The thymus of uninfected flocks showed a normal histological structure of the thymic lobules that were enclosed by a fibrous connective tissue capsule with an intact cortex and medulla, in which the cortex displayed a dense lymphocyte population, giving the cortex deep basophilic staining. Similarly, the spleen of uninfected flocks showed a normal splenic tissue that was characterized by a diffuse population of T-lymphocytes forming the peri-arteriolar lymphoid sheath with normal bursa-dependent lymphoid follicles containing B-lymphocytes. Regarding the bursa of Fabricius in the uninfected flocks, intact plica epithelium covered numerous normal lymphoid follicles separated by thin bands of fibrous connective tissue. Negative immune staining was observed in the uninfected flocks using the prepared antibody. Similarly, negative immunostaining was noticed in the infected flocks with the escaping of primary antibody addition in the immune protocol (Figure 6).

### 3.3. Conventional and Real-Time PCR Results

Detection of NGPV in a total of 75 pools (each with samples from 2 to 3 ducks per flock) of the thymus (*n* = 25), bursa of Fabricius (*n* = 25), and spleen (*n* = 25) revealed 65.3% positives (*n* = 49). Using conventional PCR, the Muscovy pooled samples (*n* = 21) yielded 9 (42.9%) positive NGPV, while Native pooled samples (*n* = 6) yielded 5 (83.3%) positive NGPV and no MDPV was detected in all samples. Real-time PCR revealed 33 NGPV-positive out of 45 Pekin pooled samples (73.3%), while Mule pooled samples revealed 2 NGPV-positive out of 3 Mule samples (66.7%). According to the results of conventional PCR and real-time PCR, the highest positive percentage of 76% (*n* = 19), was detected in the bursa, followed by 60% (*n* = 15) for each of the thymus and spleen (Table 2). The NGPV-positive real-time PCR samples were resubmitted to a discriminative conventional PCR, and the presence of NGPV was established with a total exclusion of MDPV.

### 3.4. MCA and HCA Results

**Figure 7:**
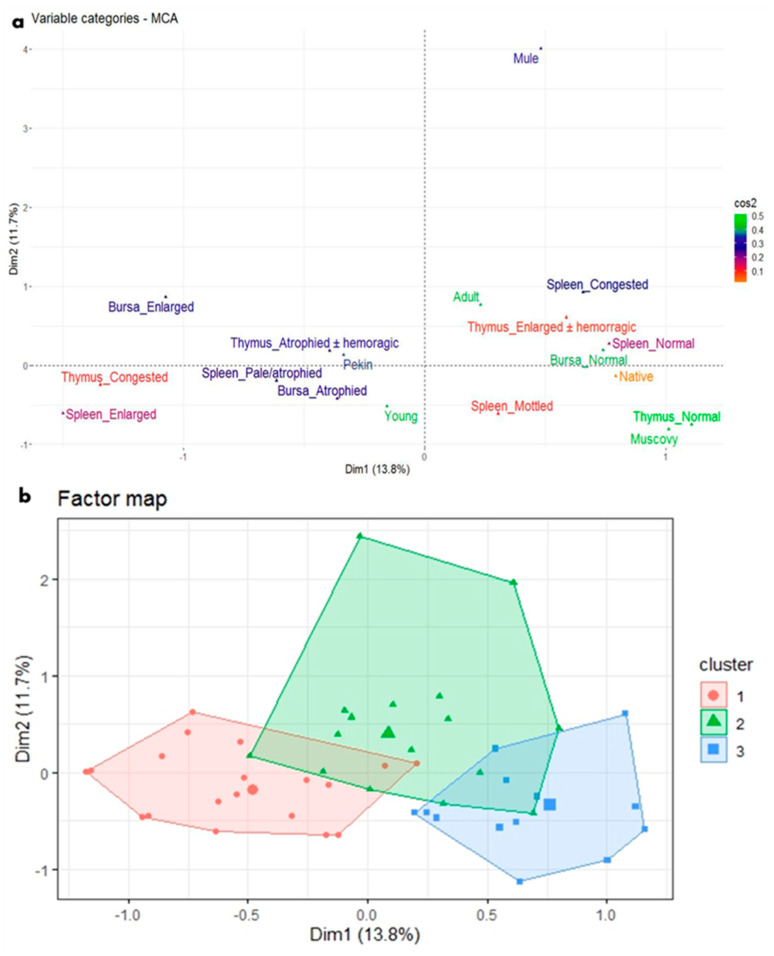
MCA and HCA of macroscopic lesions in relation to the age and breed of NGPV-infected ducks. (**a**) The MCA plot revealed the relationship between the age of ducks, duck breeds, and the recorded macroscopic lesions in the spleen, thymus, and bursa of Fabricius. The closer the points were to each other, the higher the relationship was. The points that were separate and far away considered outliers and were less frequent. (**b**) The HCA factor map of the macroscopic lesions, which were clustered into three clusters in relation to the age and breed of the examined ducks.

Regarding the macroscopic lesions in the immune system organs of the parvovirus-infected ducks, the age and breed variables were important for assessing the pattern of relationships of macroscopic lesions found in the bursa of Fabricius, spleen, and thymus of the examined ducks. Based on the age of the ducks, the reported macroscopic lesions were clearly distinguished into two poles on the MCA plot, which indicated that the lesions were exhibited in relation to the age (Figure 7a) as follows:

- Apparently normal spleen, bursa of Fabricius, and the thymus were strongly related to adult Muscovy and Native ducks and closer to each other on the MCA plot.
- Atrophy of immune system organs, including pale atrophied spleen, atrophied bursa of Fabricius, and atrophied thymus (with or without hemorrhage), was closely related to young Pekin ducks on the MCA plot.
- The less frequently recorded macroscopic lesions, including mottled, enlarged, and congested spleen, enlarged bursa of Fabricius, and congested and enlarged thymus (with or without hemorrhage), were not related to a particular age or breed on the MCA plot.

HCA analysis based on MCA provided further information about the pattern of relationships by grouping those macroscopic lesions into clusters based on similarities (Figure 7b) as follows:

- Cluster 1 (the blue cluster) could be called the cluster of apparently normal immune system organs in adult Muscovy and Native ducks.
- Cluster 2 (the red cluster) could be called the cluster of atrophy of immune system organs in young Pekin ducks.
- Cluster 3 (the green cluster) involved the less representative breed (Mule breed) and the less frequent macroscopic lesions of immune organs.

**Figure 8:**
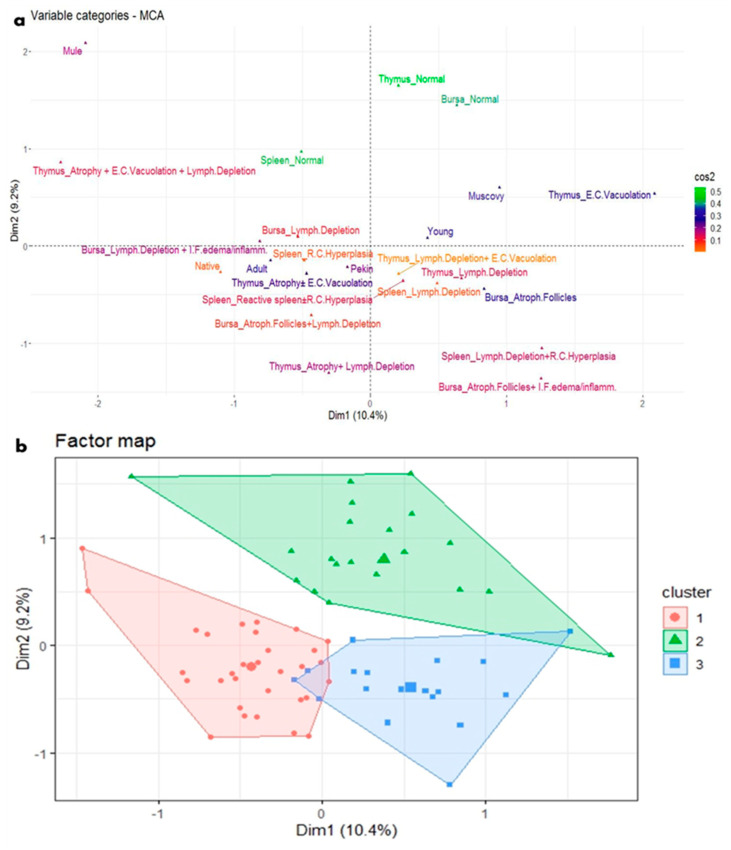
MCA and HCA of microscopic lesions in relation to the age and breed of NGPV-infected ducks. (**a**) The MCA plot revealed the relationship between the age and the recorded microscopic features in the spleen, thymus, and bursa of different duck breeds. The closer the points were to each other, the higher the relationship was. The points that are separate and far away were considered outliers and were less frequent. (**b**) The HCA factor map of the microscopic lesions, which were clustered into three clusters, in relation to the age and breed of the examined ducks.

Regarding the microscopic picture of the immune system organs of parvovirus-infected ducks, the most prominent relationships based on the MCA plot were found and summarized (Figure 8a) as follows:

- Young ducks, mainly Pekin, showed several microscopic lesions, including spleen lymphoid depletion, reactive spleen (with or without reticular cell hyperplasia), bursal atrophied follicles, and thymus lymphoid depletion (with or without epithelial cell vacuolation).
- Adult Native and Pekin ducks were mainly characterized by bursal lymphoid depletion (with or without interfollicular edema and inflammation), spleen reticular cell hyperplasia, and thymus atrophy (with or without epithelial cell vacuolation).
- Normal histological features were less likely to be found microscopically and were only observed in Muscovy ducks.

Based on HCA, three clusters are derived from the most significant relationships between categories, which are grouped together as comparable within a cluster (Figure 8b) as follows:

- The first cluster (the blue cluster) combined young Pekin ducks characterized by spleen lymphoid depletion, reactive spleen (with or without reticular cell hyperplasia), bursal atrophied follicles, and thymus lymphoid depletion (with or without epithelial cell vacuolation).
- The second cluster (the red cluster) combined adult Native and Pekin ducks characterized by bursal lymphoid depletion (with or without interfollicular edema and inflammation), spleen reticular cell hyperplasia, and thymus atrophy (with or without epithelial cell vacuolation).
- The third cluster (the green cluster) combined the category of normal histological features, which was rarely found and only predominant in the Muscovy breed.

### 3.5. NGS and Phylogenetic Analysis

Three strains identified in the current study were subjected to whole genome sequencing using NGS, named Goose-parvovirus-EgyArmy-ZU-202-2022, Goose-parvovirus-EgyArmy-ZU-239-2022 (Pekin origin), and Goose-parvovirus-EgyArmy-ZU-274-2022 (Muscovy origin), and were submitted to GenBank with accession numbers OR416224, OR416225, and OR416226. The three whole genome sequenced strains were classified as variant GPVs (NGPV) and showed up to 98.8% similarity with each other.

**Figure 9:**
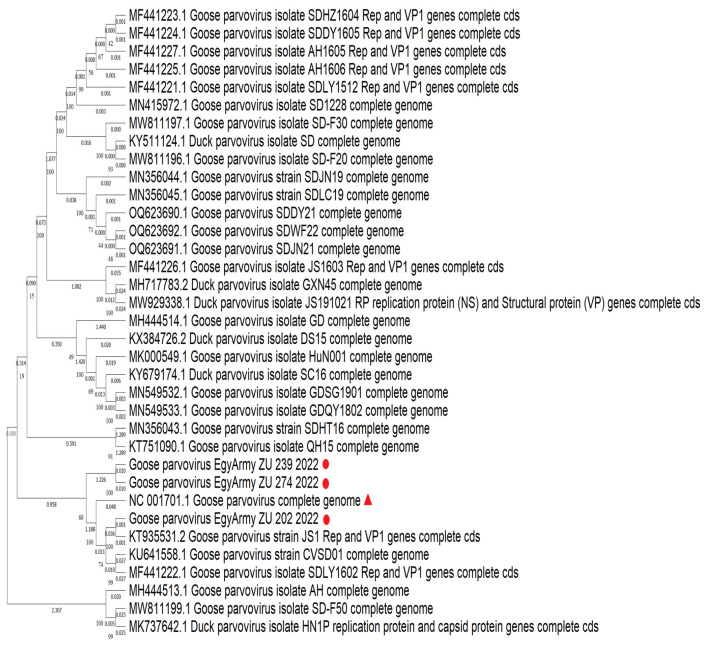
Bootstrap phylogenetic tree based on sequence alignment of whole genome sequences of NGPVs. Numbers on the branches indicate bootstrap percentages obtained using 500 replicates. Circles refer to NGPV strains determined in this study, while the triangle shape refers to the classical GPV B strain.

**Figure 10:**
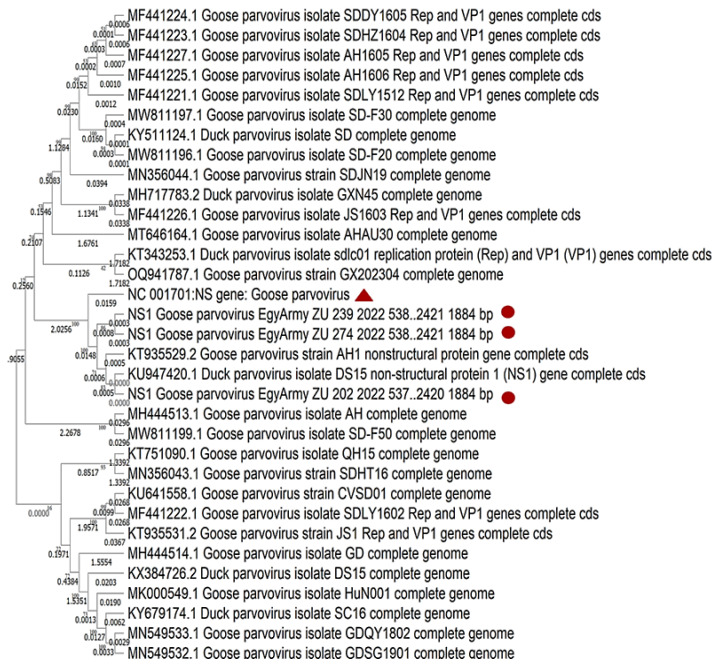
Bootstrap phylogenetic tree based on *NS* sequence alignment of NGPVs. Numbers on the branches indicate bootstrap percentages obtained using 500 replicates. Circles refer to NGPV strains determined in this study, while the triangle shape refers to the classical GPV B strain.

**Figure 11:**
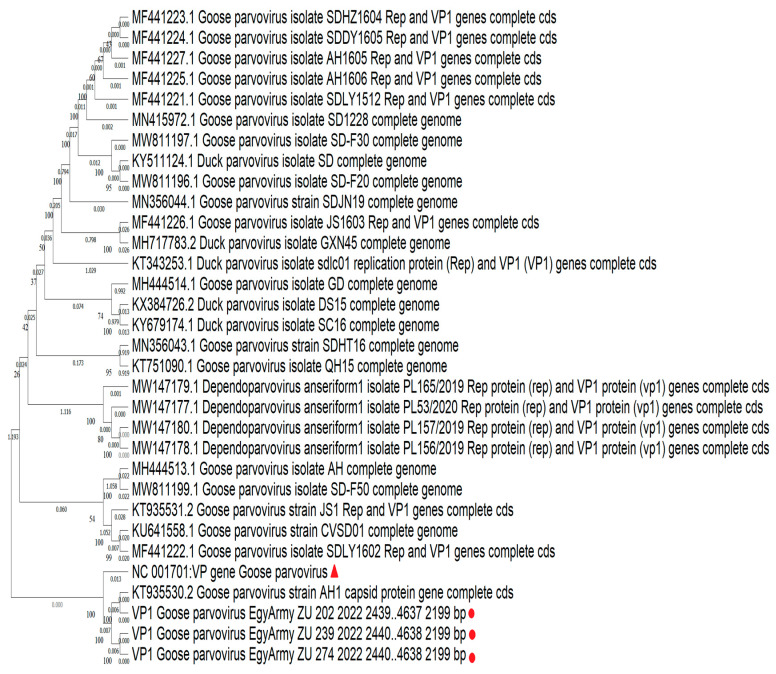
Bootstrap phylogenetic tree based on *VP* sequence alignment of NGPVs. Numbers on the branches indicate bootstrap percentages obtained using 500 replicates. Circles refer to NGPV strains determined in this study, while the triangle shape refers to the classical GPV B strain.

Using the NCBI database, 30 complete genome sequences of NGPV were found to be much closer to our isolates. Comparing the whole genome sequences of the three Egyptian NGPV strains, they were closely related to the recent Chinese isolates identified in Mainland China since 2015, as the nucleotide identity with those Chinese NGPV isolates was up to 99.57% (Figure 9). The percentages of nucleotide identity between the first identified Chinese NGPV strain (Sdlc01-Pekin isolate, 2015) and our isolated strains, GPV-EgyArmy-ZU-274-2022, GPV-EgyArmy-ZU-239-2022, and GPV-EgyArmy-ZU-202-2022, were 99.68%, 99.63%, and 99.84%, respectively on the *NS* level (Figure 10), and 98.23%, 98.18%, and 99.86%, respectively, on the *VP* level (Figure 11).

Furthermore, the inserted classical virulent GPV B strain revealed lower percentages of nucleotide identity with Egyptian NGPVs on the whole genome, *NS*, and *VP* levels, as shown in Table 3. The percentages of *VP* nucleotide identity with European vaccine strains currently used in waterfowl vaccination programs against parvovirus infection in Egypt are also shown in Table 3.

## 4. Discussion

As a continuing threat, GPV negatively affects the duck industry in Egypt, even with regular vaccination, particularly in commercial flocks. The present study aimed to identify and investigate the circulating strains of GPV and their impact on the immune system organs in different duck breeds and ages in Sharkia Province, Egypt. Twenty-five duck flocks exhibiting a clinical history of suspected GPV infection were investigated based on the clinical picture, postmortem and histopathological examination, viral genome detection, IHC, statistical analysis of risk factors affecting the frequency of macroscopic and microscopic features, and full genome sequencing.

The variation in the morbidity rate of NGPV infection among flocks could be attributed to either variation in host susceptibility between Muscovy and Pekin ducks or the presence of maternally derived antibodies (MDA) [10,13,43], comparable to the current study in which all examined ducks (except Native flocks) hatched from vaccinated breeders, so there was a partial level of protection against the severe state of the disease. This agreed with the findings of Palya et al. (2009) that observed a higher morbidity rate after NGPV experimental challenge in one-day MDA-free Mule ducklings (70%) than in one-day Mule ducklings hatched from parvovirus-vaccinated breeders (33%) [10], and also agreed with the findings of Chen et al. (2016) which recorded a high morbidity rate after NGPV experimental challenge either in Mule (80%) or Cherry Valley (90%) three-day-old MDA-free ducklings [16].

Regarding the low mortalities in the present study, Zhu et al. (2022) also recorded a low mortality rate of 1% in NGPV-naturally infected Cherry Valley ducks [44]. Also, naturally infected Mule and Cherry Valley flocks displayed a 2% mortality rate [17]. At the same time, Palya et al. (2009) recorded 3 and 5% mortality rates in NGPV-experimentally infected Mule ducklings either hatched from vaccinated or non-vaccinated breeders, respectively [10]. Even if NGPV is detectable by PCR, it can result in postmortem and histological changes in various organs without causing any mortality [10,33]. Lower mortality and smaller beaks are the major discriminators between fully susceptible, MDA-free, classical GPV, and NGPV-infected ducks. As classical GPV does not severely infect Mule and Pekin duck breeds, NGPV, as a variant GPV strain, is not completely adapted to cause severe disease in Mule and Pekin ducks, which might be the reason for the low mortality caused by this variant strain in such breeds [10,17,35,45].

In the current study, common clinical signs were recorded in examined ducks of variable ages (14–75 days old). Similarly, there was a wide spectrum of age susceptibility, as the disease was recorded in all ducks from one day old until 50 days of age [46]. Comparable clinical features have been previously reported in several publications that recorded WFPV infections over the years [1,2]. The same clinical signs, in addition to short beaks with protruding tongues, have been recorded among NGPV-infected ducks [10,13], and were also recorded in the examined Muscovy and Native flocks, except for tongue protrusion. Xiao et al. (2017) investigated the pathogenicity of the NGPV M15 strain in different duck breeds, including the Muscovy breed. Although NGPV-infected Muscovy ducklings suffered from severe growth retardation, no Muscovy ducklings showed tongue protrusion, and the decrease in beak length and width was insignificant compared to non-infected ducks [43]. Moreover, Zhu et al. (2022) profoundly compared the NGPV pathogenicity between Muscovy and Pekin breeds and recorded that both Cherry Valley-origin NGPV (CVD21) and Muscovy-origin NGPV (MD17) were able to experimentally infect Cherry Valley Pekin ducklings, causing the appearance of the typical clinical picture of SBDS, while obvious clinical signs were observed in the Muscovy ducklings infected with MD17, mainly growth retardation and decreased beak width and length, without tongue protrusion [44].

In parvovirus-infected flocks, atrophy of lymphoid tissues, including the bursa, thymus, and spleen, was recorded during the postmortem examination of clinically diseased ducks from different breeds [45,47,48]. Atrophy of the immune system organs was more prominent in Pekin ducks, which could be attributed to the relative increase in disease severity in this breed [16,33] compared to other duck breeds [43].

The histopathological findings were also previously recorded in several reports investigating WFPVs’ pathogenesis under natural or experimental circumstances, including necrotic thymic medulla with Hassel’s corpuscle disintegration [33], depletion and degeneration of thymic lymphocytes [30,47,49], disappearance of normal bursal histological morphology accompanied with atrophy of bursal lymph follicles and lymphocytic depletion [5,35,47,49], spleen degeneration characterized by lymphocytic depletion [5,30,44,47], and reticular cell proliferation [10].

Surprisingly, no previous IHC work was conducted for NGPV infection to compare with the IHC findings in the current study. Only GPV was previously detected by immunoassay in the spleen of experimentally-infected goslings [50], whereas it was detected in the bursa of Fabricius, thymus, and spleen, after the experimental challenge with GPV in Cherry Valley ducklings [30]. Histopathological and IHC findings were confirmed using molecular identification, which established the NGPV infection with the complete exclusion of MDPV infection.

The primer selection was critical for distinguishing between MDPVs and GPVs (including classical and variant strains) in the present study. Genomic comparison between GPVs and MDPVs revealed that they share 80–84% nucleotide similarity at the genome level [15,25]. These data indicate that false PCR results can be obtained if primers are not designed to target specific regions. To overcome this drawback, we used a conventional PCR assay based on *NS* phylogenetic analysis of MDPV and GPV isolates and provided an alternative tool to detect and differentiate between MDPVs and GPVs with increased accuracy [25]. We also performed a real-time PCR assay based on the characteristic variable regions of *NS* genes in GPVs and MDPVs for the specific detection of classical GPV and NGPV with the total exclusion of MDPV detection probability. Moreover, classical GPV and NGPV could be distinguished using this assay coupled with host specificity, as the samples were considered NGPV positive if they were successfully detected in Pekin and Mule ducks and considered classical GPV positive if it was successfully detected in Muscovy ducks and goslings [28]. However, this concept was dropped in our study while we confirmed the variant GPV (NGPV) in both Muscovy ducks and Pekin ducks using NGS.

The PCR assay revealed an unexpected result, as the bursa was the primary organ for NGPV detection (76%) among the examined flocks. Unfortunately, there are few available records in which NGPV was detected in the bursa in naturally-occurring NGPV outbreaks. The few records could be attributed to pooled mixed organ samples submitted for PCR detection, so the organ of detection could not be defined [51]. GPV copy numbers in the spleen, thymus, and bursa following experimental infection in Muscovy ducklings were detected as early as the first dpi until the end of the observation period [52]. Following experimental infection in goslings, GPV viral load was detected in all immune system organs [50] and was significantly higher than GPV viral load in any other organ [53]. Comparably, these immune system organs could be considered the primary sites of invasion in ducks after NGPV infection; in turn, they might greatly compromise the immune response of infected ducks, as evidenced by the abovementioned negative impact on the immune system of birds under study.

Based on the statistical analysis of the macroscopic and microscopic features to determine lesion frequency in relation to the age and breed of examined ducks using MCA and HCA, the results found in this study showed that all duck ages and breeds, organs of the immune system were negatively affected, with a variable degree of severity. In parvovirus-infected young waterfowls, atrophy of lymphoid tissues including the bursa, thymus, and spleen was recorded during pathological examination of goslings and Muscovy ducklings [47], Cherry Valley Pekin ducklings [45], Mule ducklings [48], and thymic petechial hemorrhage [48,49], as the ducklings hatched from non-vaccinated breeders. In infected young Pekin and Mule ducks, a congested, enlarged thymus with or without petechial hemorrhage [13,30,33,45] and splenomegaly [30,33] were observed, as evidenced in the present study. Although Muscovy and Native ducks were apparently normal, the microscopic picture demonstrated the immune impairment of these animals.

A typical microscopic picture of lymphoid atrophy was identified in young Pekin ducks as well as in adult Pekin and Native ducks. In previous studies, analogous histopathological changes were recorded: thymus atrophy with lymphocytes and reticular cells scattered in the necrosis, and thymus corpuscle disintegration was observed after experimental infection in NGPV-infected Cherry Valley Pekin ducklings [33], depletion and degeneration of thymic lymphocytes in GPV-infected Cherry Valley Pekin ducklings [30], disappearance of normal bursal histological morphology accompanied by atrophied bursal lymph follicles and lymphocytic depletion in NGPV-infected Pekin ducklings [35] and NGPV-infected goslings [5]. In addition, splenic lymphocyte necrosis and depletion in GPV-infected Cherry Valley ducklings [30], NGPV-infected Pekin and Muscovy ducklings [44], and reticular cell proliferation in NGPV-infected Mule ducklings [10] were also recorded and supported the findings of the present study.

The low cross-protection of breeder vaccination in examined Pekin ducklings and the absence of any applied vaccination regimen in examined adult Pekins led to microscopically severe disease at both ages, confirmed by the PCR percentage of detection (73.3%). The reason why Native ducks expressed the disease only in the adult age with 83.3% percent of PCR detection in spite of hatching from non-vaccinated breeders may be due to the natural resistance of the Native breed compared with foreign duck breeds (Pekin and Mule). Less frequent normal histological features in organs of the immune system were recorded only within Muscovy ducks, as the examined Muscovy flocks were only 42.9% PCR-positive reactors. Serious pathological changes may cause immunosuppression, resulting in an enhanced opportunity for co-infection with other viral or bacterial pathogens and potentially leading to vaccination failure, which is highly indicative of WFPV (including NGPV) tropism in the immune system [30,48,49,50,53].

Based on viral genome detection and identification, all isolates in the current study were identified as NGPVs. Phylogenetic analysis of NGPV strains in the current study revealed they were closely related to Chinese NGPV (up to 99%) isolates, as recent studies from Poland and Vietnam declared about their isolates [12,51]. Comparably, many recent phylogenetic analyses of NGPVs worldwide have dedicated a close relationship to Chinese NGPV strains with a less genetic relationship with classical GPVs [17,18,54,55]. All previously reported alignment data indicated that the similarity among NGPVs was still higher than the similarity between NGPVs and GPVs, as we declared in the present study. Gene annotation analysis of the identified NGPV strains was also within the same range as a recent genetic map for NGPV revealed [56].

Regarding European vaccine strains currently used in Egypt in vaccination programs against WFPVs, the percentage of *VP* nucleotide identity between the identified NGPV strains under the current investigation and those vaccine strains (Table 3) might be insufficient for the complete protection against circulating NGPVs and SBDS prevention. Commercially used vaccines have not included NGPV strains until now; therefore, they do not produce such great cross-protection in NGPV-infected flocks. This may explain why they were highly susceptible to infection and could justify the severe atrophy lesions in the immune system organs. Our deduction was confirmed by a comparative study in which all MDA-free Mule ducklings were GPV- and NGPV-positive PCR reactors until six weeks post-infection, regardless of the age at which the ducklings were challenged. However, ducklings from vaccinated breeders were only NGPV-positive PCR reactors until the fifth week after the challenge [10].

Consequently, it has been recommended that there is a need to evaluate the protective efficacy of classical GPV vaccines against NGPV, and to update the available commercial vaccines to include recent isolates [17]. NGPV incidence seems to be attributed to the alteration of host ranges of GPV caused by virus variation [57]. NGPV host susceptibility and evolution are gradually expanding among waterfowl, consequently leading to vaccination failure because of outdated commercial vaccines and/or defects in the vaccination regimen itself [18,43].

## 5. Conclusions

Variant GPV (NGPV) infection was proven among all duck breeds of different ages to have a bad impact on organs of the immune system, particularly in Pekin ducks, even with breeder vaccination. Disease severity is dependent on a specific immune status rather than on age susceptibility. Based on full genome sequencing, Egyptian NGPVs are closely related to recent Chinese NGPV isolates. More inclusive work investigating virus evolution, epitope mutation, pathogenicity, and immunogenicity among several recently isolated and completely identified Egyptian NGPV strains is still under current research.

## Figures and Tables

**Table 1:** Primers and Probes used in this study targeting the *NS* protein-coding gene of MDPV and GPV strains.

PCR Type	Primer Name	Nucleotide Sequence (5′-3′)	PCR bp	Reference
Conventional	MDPV-F1	GATGAATGCTGTAGTGCAGGAGGA	549	[25]
	GPV-F1	TTTGGCHGCCCCTTTACCTGATCC		
	NGPV-R	ATTTTTCCCTCCTCCCACCA		
TaqMan Real-time	GPV-qF	TAGGGAGGAGTTAGAAGA	-	[28]
	GPV-qR	TACTTATGACAATTCTATGGATG		
	GPV-qP	ACCTGGTAATTGTTCYTGCTTCTCT		

PCR, polymerase chain reaction; *NS*, nonstructural; MDPV; Muscovy duck parvovirus; GPV, goose parvovirus; NGPV, novel goose parvovirus; bp, base pair.

**Table 2:** Conventional and real-time PCR findings.

PCR Type	Breed	No. of Tested Tissue Pools	Organ	No. of NGPV-Positive Pools/No of Tested Tissue Pools	Total No. of NGPV-Positive Tissue Pools	Total No. of NGPV-Negative Tissue Pools
Real-time	Pekin	45	Bursa	14/15	33	12
			Thymus	9/15		
			Spleen	10/15		
	Mule	3	Bursa	1/1	2	1
			Thymus	1/1		
			Spleen	0/1		
Conventional	Muscovy	21	Bursa	2/7	9	12
			Thymus	3/7		
			Spleen	4/7		
	Native	6	Bursa	2/2	5	1
			Thymus	2/2		
			Spleen	1/2		
Total	-	75	-	49/75	49	26

**Table 3:** Genome annotation, the nucleotide identity of classical GPV B strain with the three Egyptian NGPVs, and the nucleotide identity of the *VP* sequences of Egyptian NGPVs with the *VP* sequences of European vaccinal strains currently used in Egypt.

Sequence_ID	Whole Genome Length (bp)	Replication Protein (*NS*)			Capsid Protein (*VP*)			Nucleotide Identity with Classical GPV B Strain			*VP* Sequence of LB Strain		*VP* Sequence of Hoekstra Strain	
		Whole Length (bp)	Start Site (bp)	End Site (bp)	Whole Length (bp)	Start Site (bp)	End Site (bp)	Whole Genome Level	*NS* Level	*VP* Level	*VP1/VP2*	*VP3*	*VP1/VP2*	*VP3*
GPV-EgyArmy-ZU-202-2022	5107	1884	537	2420	2199	2439	4637	96.28%	96.92%	96.59%	96.16%	96.97%	98.65%	98.99%
GPV-EgyArmy-ZU-239-2022	5106	1884	538	2421	2199	2440	4638	96.18%	96.76%	96.45%	96.61%	96.97%	94.58%	98.99%
GPV-EgyArmy-ZU-274-2022	5101	1884	538	2421	2199	2440	4638	96.20%	96.82%	96.50%	96.61%	96.97%	94.58%	98.99%

## Data Availability

The datasets generated for this study are freely available on Figshare with a DOI number: https://doi.org/10.6084/m9.figshare.24634338. Data of three fully identified and sequenced Egyptian NGPV strains are available on GenBank.

## Supplementary Materials

The following supporting information can be downloaded at: https://www.mdpi.com/article/10.3390/v17010096/s1, Table S1: Descriptive data of clinically examined duck flocks.
