# Supplementary material for: Egyptian Novel Goose Parvovirus in Immune Organs of Naturally Infected Ducks: Next-Generation Sequencing, Immunohistochemical Signals, and Comparative Analysis of Pathological Changes Using Multiple Correspondence and Hierarchical Clustering Approach
Source: Viruses. 2025 Jan 13;17(1):96. doi: 10.3390/v17010096 (PMC11769075; doi:10.3390/v17010096)
Supplement: Supplementary file 1 [file viruses-17-00096-s001.zip › viruses-3348383-supplementary.pdf]

Supplementary Table 1. Descriptive data of clinically examined duck flocks

Flock no.	Breed	No. of examined ducks	Age\ Day	Total flock no.	Mortality no.	Morbidity		Previous Parvovirus vaccination	Clinical findings
						No	%		
**1**	Muscovy	4	20	4000	-	50	1.25	Breeder vaccination<br><br>Live-attenuated vaccine s\c at 2 weeks of age	General weakness<br>Abnormal feathering<br>Skin redness<br>Diarrhea<br>Locomotor abnormalities<br>Growth retardation
**2**	Muscovy	2	75	1500	-	30-40	2-2.7	Breeder vaccination	General weakness<br>Abnormal feathering<br>Skin redness<br>Diarrhea<br>Growth retardation
**3**	Muscovy	3	18	1000	10-15	50	5	Breeder vaccination<br><br>Live-attenuated vaccine s\c at 2 weeks of age	Growth retardation<br>Diarrhea
**4**	Muscovy	1	70	500	10	50	10	Breeder vaccination	General weakness<br>Abnormal feathering<br>Diarrhea<br>Growth retardation
**5**	Pekin	5	35	1500	-	30-50	2-3.3	Breeder	Severe growth retardation<br>Abnormal feathering
**6**		5	35	1500	-				

**7**		5	40	1500	-			vaccination	Skin redness<br>Protruded necrotic tongue<br>Beak atrophy<br>Diarrhea
**8**		5	40	1500	-				
**9**	Native	2	40	200	-	All	100	-	Growth retardation<br>General weakness<br>Abnormal feathering<br>Skin redness<br>Locomotor disturbance
**10**	Pekin	5	42	3000	-	1500	50	Breeder vaccination	Severe growth retardation<br>Abnormal feathering<br>Skin redness<br>Protruded necrotic tongue<br>Beak atrophy<br>General weakness<br>Diarrhea
**11**	Pekin	5	42	3000	-	1000	33.3	Breeder vaccination	
**12**	Pekin	5	30	6000	-	3000	50	Breeder vaccination	Severe growth retardation<br>Abnormal feathering<br>Skin redness<br>Protruded necrotic tongue<br>Beak atrophy<br>General weakness<br>Diarrhea
**13**	Native	1	45	20	-	All	100	-	Growth retardation<br>General weakness<br>Abnormal feathering<br>Skin redness<br>Diarrhea<br>Locomotor disturbance

**14**	Mule	2	65	1500	10-15<br>At 9-15-day-old	100-150	6.7-10	Breeder vaccination	Growth retardation<br>Protruded necrotic tongue<br>Beak atrophy<br>Diarrhea
**15**	Pekin	6	20	2200	15<br>At 7-12 day-old	230-250	10.5-11.4	Breeder vaccination	Severe growth retardation<br>Protruded necrotic tongue<br>Beak atrophy<br>Diarrhea
**16**	Pekin	2	60	4000	10-15	90	2.25	Breeder vaccination	Severe growth retardation<br>Protruded necrotic tongue<br>Beak atrophy<br>Diarrhea
**17**	Pekin	7	20	2020	30<br>At 7-day old	100-150	5-7.4	Breeder vaccination	Severe growth retardation<br>Protruded necrotic tongue<br>Beak atrophy<br>Diarrhea
**18**	Pekin	5	14	1500	-	30-50	2-3.3	Breeder vaccination	Severe growth retardation<br>Short beak<br>Diarrhea
**19**	Pekin	6	14	1500	-	30-50	2-3.3	Breeder vaccination	
**20**	Pekin	6	15	2000	-	50-100	2.5-5	Breeder vaccination	Severe growth retardation<br>Short beak<br>Diarrhea
**21**	Pekin	3	15	2000	-	50-80	2.5-4	Breeder vaccination	Severe growth retardation<br>Short beak<br>Diarrhea<br>General weakness

**22**	Pekin	3	17	1500	-	50	3.33	Breeder vaccination	Severe growth retardation<br>Short beak<br>Diarrhea<br>General weakness
**23**	Muscovy	5	22	2500	-	50	2	Breeder vaccination	Severe growth retardation<br>Diarrhea<br>General weakness
**24**	Muscovy	5	22	2500	-	30	1.2	Breeder vaccination	growth retardation<br>abnormal feathering<br>Diarrhea<br>General weakness
**25**	Muscovy	1	23	1500	-	50	3.33	Breeder vaccination	growth retardation<br>abnormal feathering<br>Diarrhea<br>General weakness
